# Cardiomyocyte-derived exosomes carrying miR-181a-5p facilitate heart-brain crosstalk and exacerbate methamphetamine dependence in rats

**DOI:** 10.3389/fphar.2025.1541442

**Published:** 2025-05-14

**Authors:** Hancheng Li, Yongen Peng, Yangkai Wu, Yiling Chen, Jieyu Li, Yunbing He, Hongwu Wang, Chaohua Luo, Zhixian Mo

**Affiliations:** ^1^ Department of Pharmaceutical Engineering, School of Food and Pharmaceutical Engineering, Zhaoqing University, Zhaoqing, China; ^2^ Department of Pharmacology of Chinese Medicine, School of Traditional Chinese Medicine, Southern Medical University, Guangzhou, China; ^3^ Key Laboratory for Research and Utilization of Southern Medicine, Zhaoqing University, Zhaoqing, China; ^4^ Risk Assessment Laboratory for Agricultural Product Quality and Safety, Ministry of Agriculture and Rural Development, Zhaoqing University, Zhaoqing, China

**Keywords:** methamphetamine dependence, cardiomyocyte-derived exosomes, conditioned place preference, heart-brain crosstalk, miR-181a-5p

## Abstract

**Background:**

Methamphetamine (MA) is one of the most harmful synthetic drugs, yet the mechanisms underlying its addiction and relapse remain incompletely understood. This study investigates how cardiomyocyte-derived exosomes carrying miRNAs facilitate heart-brain crosstalk and contribute to MA dependence.

**Materials and methods:**

A conditioned place preference (CPP) model of MA dependence was established in rats. High-throughput sequencing were employed to identify candidate miRNAs in cardiac exosomes and brain tissues. Behavioral assessments, real-time PCR, nanoparticle tracking analysis, *in vivo* imaging, *in vitro* uptake assays, network pharmacology, and dual-luciferase reporter assays were used to explore the role of cardiomyocyte-derived exosomes in MA dependence.

**Results:**

MA induced significant CPP in rats. miR-181a-5p was markedly upregulated in cardiac exosomes and brain tissue, with higher levels observed in cardiac exosomes. *In vivo* biodistribution showed that cardiomyocyte-derived exosomes cross the blood-brain barrier and accumulate in the brain. *In vitro* uptake assays demonstrated that SH-SY5Y cells internalized these exosomes, leading to increased miR-181a-5p expression. Tail vein injections of miR-181a-5p-enriched exosomes enhanced MA CPP behavior in rats. Network pharmacology revealed 108 potential targets of miR-181a-5p, enriched in processes such as steroid biosynthesis, amide metabolism, and apoptosis, involving pathways related to the endoplasmic reticulum, MAPK signaling, and amyotrophic lateral sclerosis. Molecular docking identified stable interactions between MA and 12 targets, including HSP90B1, TNF, and MAP2K1, with miR-181a-5p binding to the 3′-UTR regions of these targets. Dual-luciferase assays confirmed the negative regulation of six targets by miR-181a-5p.

**Conclusion:**

This study reveals that cardiomyocyte-derived exosomes transport miR-181a-5p, facilitating heart-brain crosstalk and exacerbating MA CPP behavior in rats. These effects are mediated through the regulation of key brain targets, including HSP90B1, TNF, and MAP2K1, providing new insights into the molecular mechanisms of MA addiction and potential therapeutic targets.

## 1 Introduction

Methamphetamine (MA, Figure 2A) is a potent central nervous system (CNS) stimulant known for inducing euphoria, heightened alertness, and increased physical strength. However, chronic MA abuse results in severe health consequences, particularly affecting the nervous system, leading to cognitive deficits, neuroinflammation, and neurological impairments. These adverse effects are partly attributed to MA-induced neurotoxicity, involving dopamine depletion, oxidative stress, endoplasmic reticulum stress, mitochondrial dysfunction, activation of astrocytes and microglial cells, disrupted neuron-glia communication, axonal transport barriers, autophagy, and apoptosis (Thrash et al., 2009). A meta-analysis on the neuropsychological effects of MA revealed deficits in episodic memory, executive functioning, motor skills, language, and visual abilities (Scott et al., 2007). Furthermore, MA abuse is linked to an increased risk of neurodegenerative diseases, including Parkinson disease (PD) (Callaghan et al., 2010; Curtin et al., 2015) and Alzheimer disease (AD), where MA accelerates neurodegenerative processes and disrupts neurotransmitter homeostasis (Li et al., 2008; Shukla and Vincent, 2020). MA has also been associated with exacerbating symptoms in Huntington disease (HD) and has implications in schizophrenia and psychosis (Orikabe et al., 2011).

Emerging research highlights MA’s cardiotoxicity, where chronic exposure leads to myocardial fibrosis, hypertrophy, and heart failure (Lord et al., 2010). Approximately 68% of MA-related deaths involve cardiovascular pathology (Akhgari et al., 2017), with histological findings revealing myocytolysis, eosinophilic changes, necrosis, and cellular infiltration (Matoba et al., 1986). A recent autopsy study further confirmed increased collagen deposition and fibrotic remodeling in the myocardium of MA users compared to non-users (Abdullah et al., 2020). These cardiac effects highlight MA’s systemic toxicity, which can compromise the body’s circulatory system and impact critical structures such as thse blood-brain barrier (BBB). MA’s lipophilic nature enables it to cross the BBB efficiently (Davidson et al., 2022), leading to vascular toxicity and BBB disruption (Chiang et al., 2019). This damage is associated with hippocampal injury and cognitive dysfunction (Bowyer and Ali, 2006), further exacerbating MA’s neurotoxic effects, including gray matter reduction and cortical volume alterations (Orikabe et al., 2011).

Exosomes, extracellular vesicles secreted by various cells, including cardiomyocytes, play a pivotal role in intercellular communication. These vesicles carry bioactive molecules, such as miRNAs, which are involved in normal brain function and neurodegenerative diseases (Properzi et al., 2015). Certain miRNAs, particularly those enriched in the brain, have been implicated in addiction-related behaviors. For instance, MA alters miRNA expression in key addiction-related brain regions (Sim et al., 2017; Zhu et al., 2015), with miR-124 and miR-181a identified as crucial regulators of such behaviors (Bosch et al., 2015). Our prior studies also demonstrated altered miRNA expression in the hearts of MA-exposed rats, suggesting potential heart-brain crosstalk via miRNA-containing cardiomyocyte-derived exosomes (Zhou et al., 2020; Zhou et al., 2021). These findings underscore the critical role of cardiomyocyte-derived exosomes in mediating systemic responses to MA.

**FIGURE 1:**
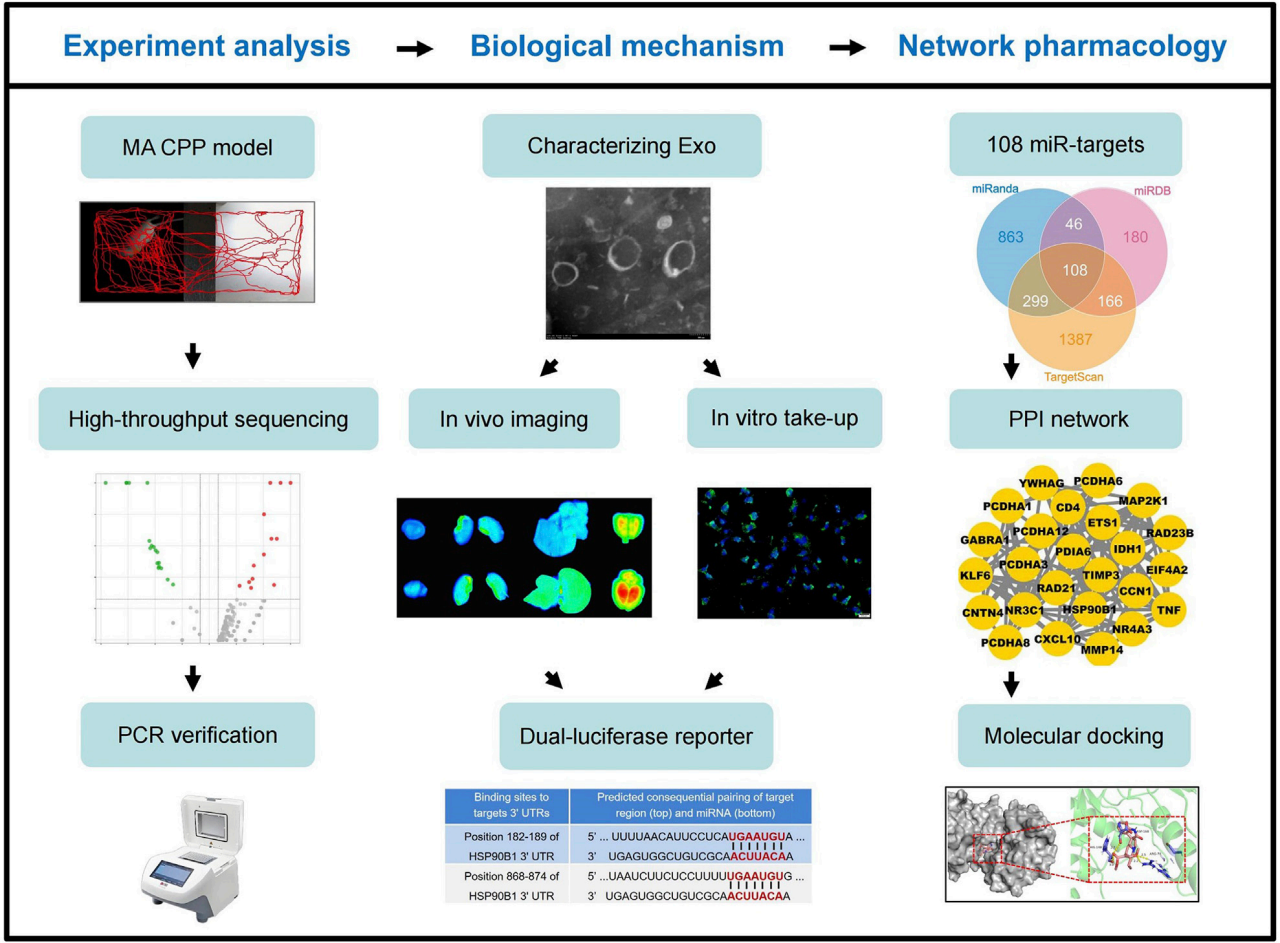
Workflow diagram of this study. MA, methamphetamine; CPP, conditioned positional preference; Exo, (cardiomyocytes-derived) exosomes; miR, miR-181a-5p.

Despite significant progress, the molecular and cellular mechanisms underlying MA-induced neurotoxicity remain inadequately understood. This study focuses on cardiomyocyte-derived exosomes carrying miR-181a-5p and their role in MA dependence. We hypothesize that MA exposure increases the release of miR-181a-5p-enriched exosomes from cardiomyocytes, which cross the BBB and accumulate in the brain. These exosomes potentially exacerbate addictive behaviors by modulating key molecular targets and signaling pathways within the brain. To test this hypothesis, we utilized a rat model of conditioned place preference (CPP) to evaluate the influence of cardiac exosome-derived miR-181a-5p on addiction-related behaviors. Our approach integrates miRNA high-throughput sequencing, exosome tracking, and network pharmacology to elucidate the mechanisms through which cardiac exosomes contribute to MA dependence (Figure 1). This research aims to deepen the understanding of peripheral signals in central addiction mechanisms and identify potential therapeutic targets for mitigating MA addiction.

## 2 Materials and Methods

### 2.1 Chemicals and biological materials

MA hydrochloride (size: 20 mg per vial; purity ≥98%; lot number 1212–9802) was procured from the National Narcotics Laboratory of China. The CPP apparatus were acquired from Shanghai Jiliang Software Technology Co., Ltd. (Shanghai, China). A rat CPP behavioral analysis system was obtained from Noldus, Information Technology, Wageningen, Netherlands. SH-SY5Y cell was purchased from Shanghai Guandao Bioengineering Co., Ltd. (Shanghai, China). DMEM/F12 medium, fetal bovine serum, heat-inactivated horse serum, trypsin, penicillin and streptomycin were provided by Gibco (Grand Island, United States). Agilent 2100 Bioanalyzer (Agilent Technologies, California, United States). Illumina Novaseq 6000 sequencing platform (Illumina, California, United States). Small Animal Live Imaging System (FX PRO, Bruker Corporation, United States). SYBR^®^ PrimeScript miRNA RT-PCR Kit was obtained from TaKaRa (Dalian, China). MiRNeasy Mini Kit was sourced from QIAGEN (Hilden, Germany). The dual-luciferase reporter assay system was provided by Promega Corporation (Madison, WI, United States). The QuikChange Site-Directed Mutagenesis Kit (11668-019) was obtained from Agilent Technologies (United States). Lipofectamine^®^ 3000 reagent was acquired from Thermo Fisher Scientific, Inc. (United States). The miR-NC, miR-181a-5p inhibitor, mimic NC, and miR-181a-5p mimic, as well as sh-RNA and shRNAs targeting HSP90B1, RAD21, MAP2K1, TNF, TIMP3 and RAD23B were all purchased from RiboBio Biotechnology Co., Ltd. (Guangzhou, China).

### 2.2 MA-dependent CPP model

Specific-pathogen-free (SPF) Sprague-Dawley (SD) rats, male, weighing 190–210 g (2 months old), were obtained from the Experimental Animal Center of Southern Medical University. The rats were housed under controlled conditions with standard chow and water provided *ad libitum*. The animal room was maintained at a temperature of 24°C–26°C, a humidity of 45%–55%, and a light/dark cycle from 8:00 a.m. to 8:00 p.m. All experimental protocols were approved by the Animal Ethics Committee of Southern Medical University (Approval No.: 20220039). A CPP rat model was established in accordance with previous studies (Li et al., 2024; Zhou et al., 2012). Based on our preliminary experiments and prior studies (Jiang et al., 2023; Li et al., 2018), a dose of 2.0 mg/kg MA was sufficient to reliably induce CPP in rats, confirming successful model establishment. Therefore, this study utilized only the 2.0 mg/kg dose for CPP induction. MA was dissolved in physiological saline to prepare a 0.4 mg/mL solution. The injection volume for each rat was calculated individually according to body weight using the formula: Dose (mg/kg) × Body Weight (kg) = Total MA dose (mg); for example, a 200 g rat received a 1 mL injection.

**FIGURE 2:**
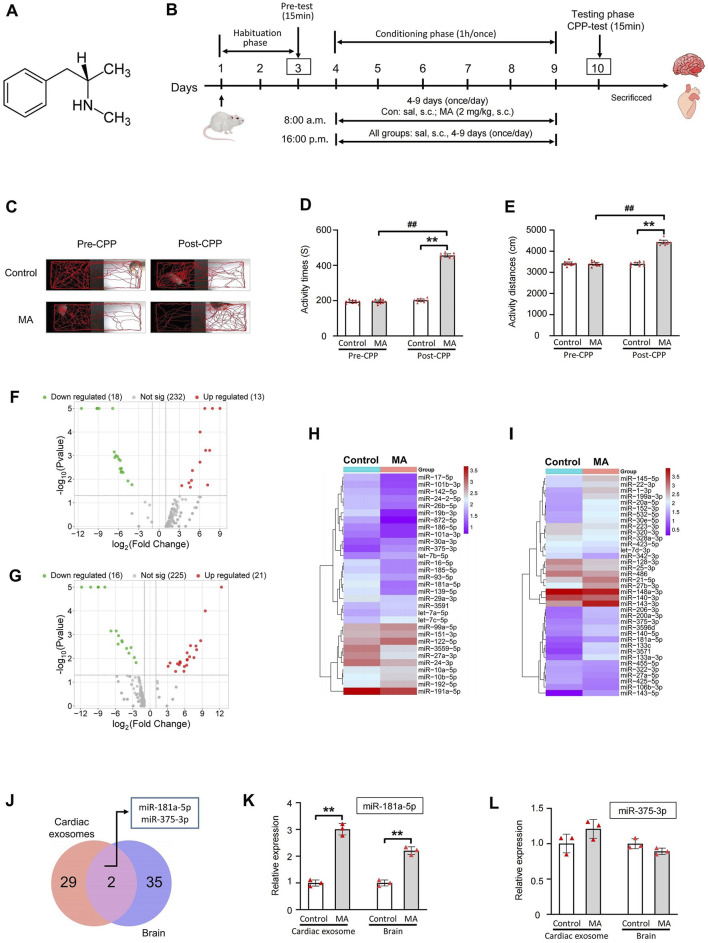
miRNAs high-throughput sequencing of cardiac exosomes and brain in MA-induced CPP rat and PCR validation of miR-181a-5p and miR-375-3p. **(A)** Chemical structure of MA. **(B)** Schematic protocol of the model of MA-dependent rats established by CPP. **(C)** Activity trajectories of rats in CPP box. Activity time **(D)** and distances **(E)** of rats in white side of CPP box, n = 10 per group. Volcano plots of expressed miRNAs of cardiac exosomes **(F)** and brain **(G)** between control and MA group. Clustering analysis of differentially expressed miRNAs of cardiac exosomes **(H)** and brain **(I)** between control and MA group. **(J)** 2 miRNAs were differentially expressed among cardiac exosomes and brain. PCR verification of differential expression of miR-181a-5p **(K)** and miR-375-3p **(L)**, n = 3 per group. ^**^p < 0.01, ^*^p < 0.05 versus the control group; ^##^p < 0.01, ^#^p < 0.05 versus the MA model group. Pre-CPP, before methamphetamine training. Post-CPP, after methamphetamine training. CPP, conditioned positional preference; MA, methamphetamine; sal, saline; s.c., subcutaneous injection.

SD rats were randomly divided into two groups (n = 10 per group): a control group and an MA model group (2.0 mg/kg). The CPP protocol consisted of three phases conducted over 10 days (Figure 2B). During the habituation phase (days 1–3), each rat was allowed to freely explore both the black and white compartments for 30 min per day. On day 3, a 15-min session was recorded on video. Rats exhibiting abnormal behavior during the Pre-CPP test were excluded from further analysis. In the conditioning phase (days 4–9), rats in the MA model group received a subcutaneous injection of MA (2.0 mg/kg) each morning at 8:00 a.m., while the control group received an equivalent volume of physiological saline. Immediately after the injection, rats were confined to the white compartment for 1 h. After an 8-h interval (at 4:00 p.m.), all rats were injected with saline and confined to the black compartment for another hour. On day 10, during the testing phase, rats were placed in the CPP apparatus for a 15-min trial with free access to both compartments. The time spent and total distance traveled in the white compartment were recorded. locomotor activity and spatial preference were analyzed using Noldus EthoVision XT 8.5 software.

Following completion of the CPP test, rats were deeply anesthetized via intraperitoneal injection of sodium pentobarbital (50 mg/kg). Euthanasia was performed only after confirming the absence of reflex responses to ensure full unconsciousness. Once anesthesia was confirmed, rats were sacrificed by transcardial perfusion with PBS, followed by 4% paraformaldehyde for tissue fixation. All procedures involving animals were conducted in accordance with the National Institutes of Health Guide for the Care and Use of Laboratory Animals (NIH publication #85-23, revised 1985).

### 2.3 miRNA high-throughput sequencing

After CPP experiment, the cardiac and brain tissues of rats were harvested for subsequent exosome isolation and miRNA high-throughput sequencing. Total RNA, including miRNA, was extracted using the MiRNeasy Mini Kit (QIAGEN, Hilden, Germany). RNA integrity was evaluated with the Agilent 2200 TapeStation, with a RIN value of ≥7 considered acceptable. Purity was assessed based on A260/A230 (≥1) and A260/A280 (≥1.5) ratios. The NEBNext Small RNA Library Prep Set for Illumina was used to construct the small RNA library. Agarose gel electrophoresis was performed, and PCR products of 140–160 bp were isolated and purified to generate the small RNA library. Library quality was validated using the Agilent 2100 Bioanalyzer, and sequencing was conducted on the Illumina Novaseq 6000 platform. miRNA expression levels were normalized to transcripts per million (TPM) using the formula: TPM = (miRNA read count/total read count) × 10^6^. Differentially expressed miRNAs were identified using a threshold of |log_2_(Fold Change)| > 1 and P < 0.05 (Cheng et al., 2016). Subsequently, cluster analysis of the differentially expressed miRNAs was performed, and a volcano plots and clustering heatmap were generated. The miRNA high-throughput sequencing was performed with support from RiboBio Biotechnology Co., Ltd. (Guangzhou, China).

### 2.4 Culture and identification of primary cardiomyocytes

Due to the limited quantity of exosomes extracted from rat cardiac tissue, which was insufficient for further animal and cell experiments, we cultured and characterized primary cardiomyocytes. Exosomes were then isolated from these primary cardiomyocytes for subsequent experiments. The hearts of neonatal rats were rapidly removed and cut into 0.5 mm^3^ fragments. The tissue was digested with trypsin, followed by digestion with a mixture of collagenase type II and trypsin, and then the digestion was terminated. The suspension was filtered and centrifuged at 1,200 rpm. The cells were resuspended and plated in culture flasks for differential adhesion. After 90 min, the non-adherent cells were collected, centrifuged at 1,000 rpm, and resuspended in DMEM/F12 containing 10% fetal bovine serum to an appropriate concentration before seeding into culture plates.

To assess primary cardiomyocyte purity, α-Actin staining was performed. Adherent cardiomyocytes were washed and fixed with paraformaldehyde, and then blocked with goat serum containing 0.3% Triton X-100. After drying, cells were incubated with an α-Actin antibody (1:500). The following day, cells were equilibrated, then antibody was removed, and cells were washed. TRITC-conjugated secondary antibody (1:1,000) was applied and incubated in the dark. After three PBS washes, nuclei were stained with DAPI and followed by three additional PBS washes. Fluorescence was observed using a fluorescence microscope.

### 2.5 Characterization of primary cardiomyocyte-derived exosomes

#### 2.5.1 Extraction of exosomes

Primary cardiomyocyte-derived exosomes were extracted using a standard differential ultracentrifugation method (Ou et al., 2018; Théry et al., 2006). Cell culture supernatant was collected and placed in PC-thick wall centrifuge tubes. After sequential centrifugation steps at 300 g for 10 min, 2,000 g for 10 min, 10,000 g for 30 min, and 120,000 g for 70 min, the supernatant was discarded. The pellet was resuspended in an appropriate volume of PBS and subjected to a final ultracentrifugation at 120,000 g for 70 min. The resulting pellet was resuspended in 20 µL of PBS to obtain the exosome-containing solution.

#### 2.5.2 Transmission electron microscopy (TEM)

TEM was used to observe the structure of extracted exosomes (Bao et al., 2024). A diluted exosome solution was applied to carbon-coated copper grids and allowed to settle for 2 min, after which excess liquid was blotted away. The grids were then stained with 20 µL of 3% phosphotungstic acid for 2 min, excess stain was blotted off, and the grids were washed with distilled water once. After drying at room temperature for 15–20 min, samples were examined under an electron microscope at 80 kV.

#### 2.5.3 Nanoparticle tracking analysis (NTA)

NTA was employed to determine the exosome particle size (Mehdiani et al., 2015). An aliquot of the exosome solution was diluted with 1 mL of PBS and mixed thoroughly. The sample was then injected slowly and steadily into the sample chamber using a 1 mL syringe, taking care to avoid bubble formation. The solution was diluted to achieve 20–30 particles per field of view, and the instrument recorded and generated a nanoparticle size distribution report.

### 2.6 Biodistribution of PKH26-exosome in rats

An aliquot of the exosome solution was mixed with Diluent C and dispersed thoroughly. One milliliter of 2× exosome solution was combined with 1 mL of 2× PKH26 dye (1:1) and immediately mixed by pipetting to ensure uniform staining. The mixture was incubated at 25°C for 4 min, with periodic inversion of the centrifuge tube every minute to enhance staining. Staining was halted by adding 1% BSA, and the sample was left at room temperature for 1 min. Exosomes were then extracted from the staining solution using ultracentrifugation, and the pellet was resuspended in PBS to obtain PKH26-labeled exosomes.

Twenty male SD rats were obtained from the Experimental Animal Center of Southern Medical University. Specific details about the rats and their housing conditions are provided in Section 2.2 *MA-dependent CPP model*. SD rats were randomly divided into a control group and a Con+Exo 1.0 mg/kg group. The groups were intravenously injected with an equal volume of saline or 1.0 mg/kg PKH26-exosome, respectively. One hour later, the rats were euthanized, and their brains, hearts, livers, and kidneys were collected. The distribution of the fluorescently labeled exosomes in various organs was observed using the Bruker multi-mode small animal imaging system FX Pro, and images were captured.

### 2.7 Uptake assay of PKH26-exosome in SH-SY5Y cell

The SH-SY5Y cell line, derived from the SK-N-SH neuroblastoma cell line and known for its neurite-like extensions, is commonly used in MA-related studies (Chetsawang et al., 2012; Langsdorf and Chang, 2011). 3.0 μg/mL PKH26-exosomes were diluted in complete culture medium, filtered through a 0.22 µm pore filter, and added to SH-SY5Y cells. The cells were incubated in the dark at 37°C for 12 h. Following incubation, the medium was discarded, and the cells were washed three times with PBS to remove non-internalized exosomes. Cells were fixed with 4% paraformaldehyde for 30 min and then washed three times with PBS. DAPI was used to stain the nuclei, and the cells were incubated at room temperature for 10 min before washing three times with PBS. Fluorescence distribution and images were observed and captured using a fluorescence microscope.

### 2.8 Effect of exosome internalization on miR-181a-5p expression in SH-SY5Y cell

The MA-induced SH-SY5Y cell model was established following the protocol from our previously published study (Li et al., 2024). SH-SY5Y cells were randomly divided into six groups: control, MA (100 μmol/L), Con + Exo 1.5 μg/mL, Con + Exo 3.0 μg/mL, MA + Exo 1.5 μg/mL, and MA + Exo 3.0 μg/mL, with three replicates per group. The control group received an equal volume of complete culture medium. The MA model group was treated with culture medium containing 100 μmol/L MA. For the Con + Exo 1.5 μg/mL, Con + Exo 3.0 μg/mL, MA + Exo 1.5 μg/mL, and MA + Exo 3.0 μg/mL groups, cells were pre-incubated with 1.5 μg/mL or 3.0 μg/mL exosomes for 15 min, followed by the addition of either complete culture medium or 100 μmol/L MA-containing medium. After treatment, all groups were cultured for an additional 48 h, during which cell morphology was photographed. The expression levels of miR-181a-5p were then quantified using Real-time PCR.

### 2.9 Effect of tail vein injection of exosomes on the MA-dependent rat CPP model

Sixty male SD rats (180–220 g, 6–8 weeks old) were obtained from the Experimental Animal Center of Southern Medical University. All animals were housed under standard laboratory conditions (12-h light/dark cycle, temperature 22°C ± 2°C, humidity 50%–60%) with *ad libitum* access to food and water. Based on our preliminary findings, two effective doses of cardiomyocyte-derived exosomes were selected for the study: 0.5 mg/kg and 1.0 mg/kg. The MA-induced CPP model was established as illustrated in Figure 4A.

Rats were randomly assigned to six experimental groups (n = 10 per group) as follows: Control group: received subcutaneous (s.c.) injections of saline at 8:00 a.m. and 4:00 p.m. (Days 4–9), followed by tail vein injection (tv.i.) of saline at 8:00 p.m. (Days 5–9); MA group: received 2.0 mg/kg MA (s.c.) at 8:00 a.m. and saline (s.c.) at 4:00 p.m. (Days 4–9), followed by saline (tv.i.) at 8:00 p.m. (Days 5–9); Control + Exo 0.5 mg/kg group: received saline (s.c.) at 8:00 a.m. and 4:00 p.m. (Days 4–9), followed by 0.5 mg/kg exosomes (tv.i.) at 8:00 p.m. (Days 5–9); Control + Exo 1.0 mg/kg group: received saline (s.c.) at 8:00 a.m. and 4:00 p.m. (Days 4–9), followed by 1.0 mg/kg exosomes (tv.i.) at 8:00 p.m. (Days 5–9); MA + Exo 0.5 mg/kg group: received 2.0 mg/kg MA (s.c.) at 8:00 a.m. and saline (s.c.) at 4:00 p.m. (Days 4–9), followed by 0.5 mg/kg exosomes (tv.i.) at 8:00 p.m. (Days 5–9); MA + Exo 1.0 mg/kg group: received 2.0 mg/kg MA (s.c.) at 8:00 a.m. and saline (s.c.) at 4:00 p.m. (Days 4–9), followed by 1.0 mg/kg exosomes (tv.i.) at 8:00 p.m. (Days 5–9). On Day 10, all rats underwent a 15-min CPP test session. During this session, the time spent, total distance traveled, and locomotor trajectory within the MA-paired compartment (white side) were recorded and analyzed using EthoVision XT 8.5 software (Noldus Information Technology).

### 2.10 Real-time PCR

Real-time PCR was used to measure the expression levels of miR-181a-5p and miR-375-3p in cardiac or cardiomyocyte exosomes, brain, and SH-SY5Y cells. Total RNA was extracted from exosomes, brain, and SH-SY5Y cells using the miRNeasy Mini Kit (QIAGEN) according to the manufacturer’s instructions. Reverse transcription primers for miR-181a-5p, miR-375-3p, and U6 (the internal control) were synthesized by Bao Biological Co., Ltd. using the stem-loop method (primer sequences are shown in Table 1). In a 96-well thermal cycler, RNA was reverse-transcribed to cDNA with the following protocol: 50°C for 60 min, followed by 85°C for 5 s. Real-time PCR was performed using the SYBR^®^ PrimeScript miRNA RT-PCR Kit in a Stratagene Mx3005P qPCR System with the following cycling conditions: 40 cycles of 95°C for 5 s and 60 °C for 30 s, preceded by an initial 95°C step for 10 s. Each sample was run in triplicate, and results were averaged. Expression levels were calculated using the Formula 2^−ΔΔCt^, with the control group values set to 1.

**TABLE 1:** The PCR primer sequence of miR-181a-5p and miR-375-3p.

miRNA	PCR primer (from 5′ to 3′)
miR-181a-5p	F: 5′-GTCGTATCCAGTGCAGGGTCCGAGGTATTCGCACTGGATACGACACTCAC-3′ R: 5′-AACATTCAACGCTGTCGGTG-3′
miR-375-3p	F: 5′-GTCGTATCCAGTGCAGGGTCCGAGGTATTCGCACTGGATACGACTCACGC-3′ R: 5′-TTTGTTCGTTCGGCTCGCGT-3′
U6	F: 5′-CTCGCTTCGGCAGCACAAACGCTTCACGAATTTGCGT-3′ R: 5′-AACGCTTCACGAATTTGCGT-3′

### 2.11 Network pharmacological analysis of miR-181a-5p

Target genes for miR-181a-5p were predicted using TargetScan (https://www.targetscan.org/), miRDB (https://mirdb.org/), and miRanda (http://www.microrna.org/microrna/home.do). A Venn diagram identified common target genes among these databases using Venny 2.1 (https://bioinfogp. cnb.csic.es/tools/venny/). GO and KEGG analyses were performed using the DAVID database (https://david.ncifcrf.gov/). GO analysis annotated target proteins from the perspectives of biological process (BP), cellular component (CC), and molecular function (MF). Enrichment maps for GO and KEGG were generated using the Microbiology Letter platform (http://www.bioinformatics.com.cn/). A network file was created to organize miR-181a-5p and its targets, and visual network diagrams were constructed using Cytoscape 3.9.1 (https://cytoscape.org/). Protein-protein interaction (PPI) networks were obtained from STRING (https://string-db.org/) and cluster modules were analyzed using the MCODE plugin to highlight miR-181a-5p-disease associations and support further analysis.

### 2.12 Molecular docking

The structural files of the protein and the small molecule MA were retrieved from the PDB database (http://www.pdb.org) and PubChem (https://pubchem.ncbi.nlm.nih.gov/) (Berman et al., 2000). Protein structures were prepared using AutoDock, including the removal of water molecules and the addition of hydrogen atoms, and saved in the pdbqt format (Mohamed A et al., 2019). Small molecules were also hydrogenated, torsional bonds were assigned, and the structures were saved in pdbqt format. The docking grid and box were defined using Autogrid, and grid box information was exported after removing the small molecules. Molecular docking was then performed using AutoDock Vina (Song et al., 2020; Clyne et al., 2020). Binding conformations were evaluated based on the principle that lower binding energy indicates a more stable structure. Docking results were visualized with PyMOL (Jerine et al., 2021).

### 2.13 Dual-luciferase reporter assay

Potential binding sequences of miR-181a-5p to the target genes HSP90B1, RAD21, MAP2K1, TNF, TIMP3 and RAD23B were predicted using Gene Cards (https://www.genecards.org/). The wild-type (WT) and mutant-type (MT) 3′-untranslated regions (3′-UTRs) of these 6 target genes were cloned into the pmiR-RB-Report™ dual-luciferase reporter plasmid vector. Site-directed mutagenesis was performed on the miR-181a-5p binding regions within the 3′-UTRs of the target genes using the QuikChange Site-Directed Mutagenesis Kit (Stratagene; Agilent Technologies, USA) according to the manufacturer’s instructions. HEK293T cells were co-transfected with WT or MT constructs, miR-181a-5p mimics/mimic NC, and a Renilla luciferase plasmid using Lipofectamine^®^ 3000 (Invitrogen; Thermo Fisher Scientific, Inc.). After 48 h of transfection, cells were lysed and centrifuged at 12,000 rpm for 1 min to collect the supernatant. Each experimental group included three replicates. The luciferase activity was measured using a dual-luciferase reporter assay system (Promega, Madison, WI, United States), with firefly luciferase activity normalized to Renilla luciferase activity. A decrease in relative luciferase activity indicated that miR-181a-5p bound to the target gene mRNAs. This assay was conducted with assistance from RiboBio Biotechnology Co., Ltd. (Guangzhou, China).

### 2.14 Statistical analysis

All data are presented as mean ± SD, with each experiment performed at least three times independently. Statistical analyses were conducted using IBM SPSS Statistics 27.0 software and bar graphs were created by Prism 10.0 software. The differences between groups were tested using one-way ANOVA analysis of variance. A *p*-value of <0.05 was considered statistically significant.

## 3 Results

### 3.1 MA-induced CPP effect and upregulated expression of miR-181a-5p in rat cardiac exosomes and brain

We successfully established a MA dependence model in rats using CPP induced by 2.0 mg/kg MA (Figure 2B). The activity trajectories of the rats were monitored and recorded (Figure 2C). Prior to CPP training, both the MA and control groups exhibited comparable activity trajectories in the MA-paired compartment (white box). Following CPP training, the MA group demonstrated a marked increase in activity trajectories compared to the control group. Quantitative analysis revealed no significant differences in activity time (*P* = 0.732) or distance traveled (*P* = 0.727) within the white box between the two groups before CPP training (Figures 2D,E). However, after CPP training, the MA group exhibited a significant increase in activity time (*P* < 0.01) and distance (*P* < 0.01) in the MA-paired compartment compared to the control group. These results confirm the successful establishment of the CPP model and indicate that MA effectively induces a CPP response in rats.

To investigate the miRNAs involved in MA dependence, we analyzed the miRNA expression profiles in rat cardiac exosomes (Figure 2F) and brain tissues (Figure 2G). A total of 31 miRNAs were differentially expressed in the cardiac exosomes of the MA group compared to the control group (|log_2_(Fold Change)| > 1 and P < 0.05), with 18 miRNAs downregulated and 13 miRNAs upregulated (Figure 2H). In the brain, 37 miRNAs were differentially expressed between the MA and control groups, with 16 miRNAs downregulated and 21 miRNAs upregulated (Figure 2I). Further screening revealed that 2 miRNAs, including miR-181a-5p and miR-375-3p, were differentially expressed among the two groups and tissues (Figure 2J). Both miRNAs show a similar trend in cardiac exosomes and the brain, with a significant upregulation observed after MA induction. Additionally, their expression levels in cardiac exosomes are notably higher than those in the brain (Table 2).

**TABLE 2:** Expression ratio of miR181a and miR-375-3p in rat cardiac exosomes and brain.

miRNA ID	Tissue	Ratio (MA vs. Control)	Up/down
miR-181a-5p	Cardiac exosome	3.10	up
miR-375-3p	Cardiac exosome	2.32	up
miR-181a-5p	Brain	2.01	up
miR-375-3p	Brain	1.73	up

Differentially expressed miRNAs, with *P* ≤ 0.05 and |log_2_(Fold Change)| > 1.

The expression of miR-181a-5p and miR-375-3p was verified by real-time PCR in rat cardiac exosomes and brain. As demonstrated in Figures 2K,L, the expression of miR-181a-5p was markedly upregulated in the MA/Control group both in cardiac exosomes (*P* < 0.01) and the brain (*P* < 0.01), whereas miR-375-3p (*P* = 0.062 and *P* = 0.264) did not show significant changes. Intriguingly, we found that miR-181a-5p was closely associated with drug dependence in these differentially expressed miRNAs (Chandrasekar and Dreyer, 2009; 2011; Zhang et al., 2016; Wang et al., 2021) and was enriched in rat brain, which was consistent with previous observations that amphetamine remarkably increased the expression of miR-181a in the brain (Saba et al., 2012). Owing to the growing body of evidence concerning the critical role of miR-181a in drug dependence, we paid attention to miR-181a-5p for advanced study.

### 3.2 Primary cardiomyocyte-derived exosomes carrying miR-181a-5p mediate heart-brain crosstalk

**FIGURE 3:**
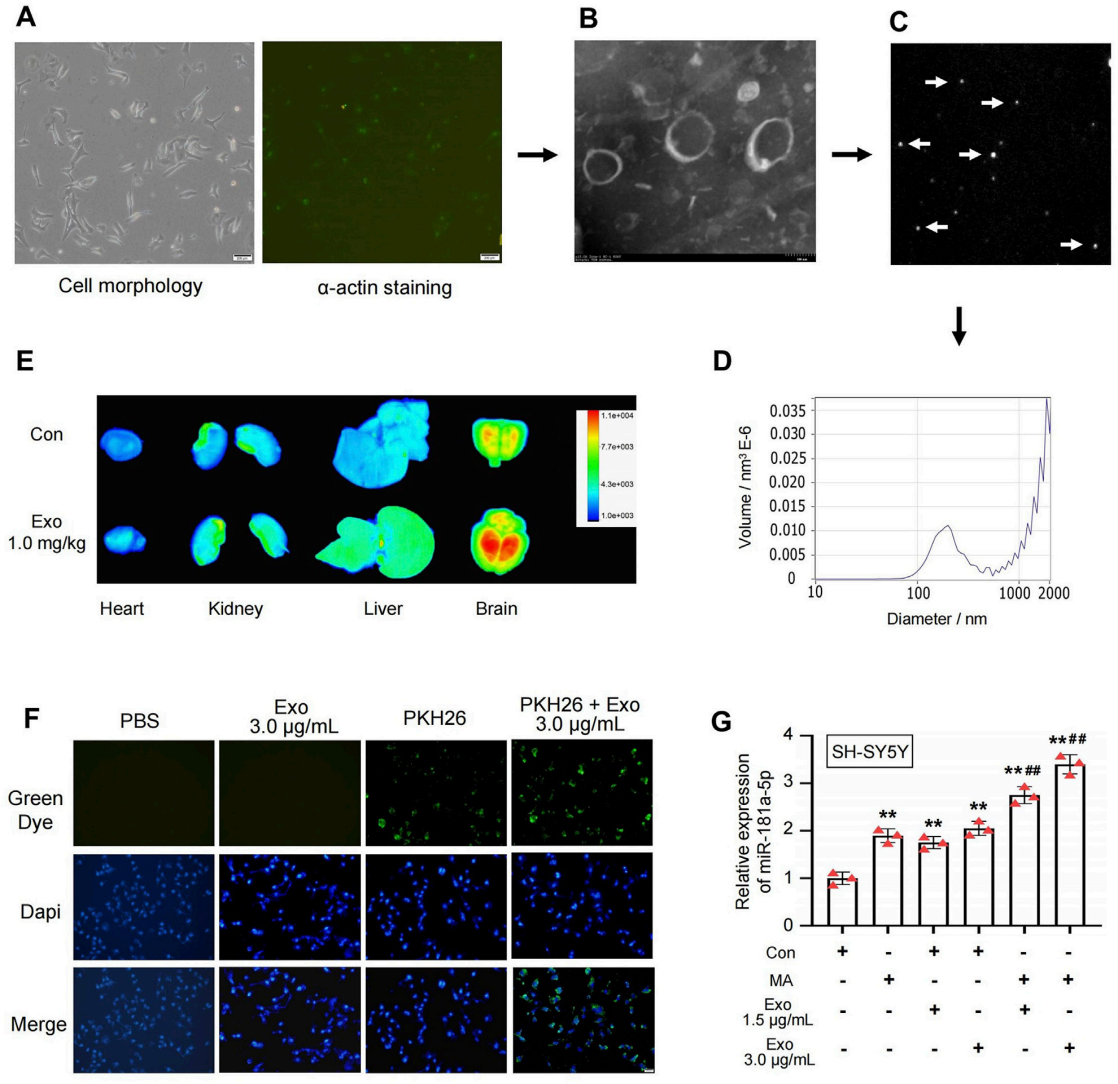
Characterization of exosomes from primary cardiomyocytes, *in vivo* imaging, and *in vitro* uptake experiments of PKH26-exosomes. **(A)** Morphology of primary cardiomyocytes and identification of α-Actin staining. **(B)** Transmission electron micrograph of exosomes. **(C)** Dynamic nanoparticle scattered light signals from exosomes were detected by nanoparticle tracking, arrows indicate nanoparticles in Brownian motion. **(D)** Nanoparticle tracking to detect the range of particle size distribution of exosomes. **(E)** After 1 h of tail vein injection with PKH26-exosomes, fluorescence expression in rat organs (heart, kidney, liver, brain) was detected by *in vivo* imaging system FX Pro. **(F)** Primary cardiomyocytes-derived exosomes can be uptaked by SH-SY5Y. **(G)** PCR detected the expression of miR-181a-5p in SH-SY5Y which were induced with MA and exosomes, n = 3 per group. ^**^p < 0.01, ^*^p < 0.05 versus the control group; ^##^p < 0.01, ^#^p < 0.05 versus MA group. MA, methamphetamine; Con, Control; Exo, Exosome.

Due to the insufficient quantity of exosomes isolated from rat cardiac tissue to meet the experimental needs, primary cardiomyocytes, which closely resemble cardiac tissue physiology, were cultured and identified in this study. Subsequently, exosomes from these primary cardiomyocytes were extracted for further experiments. As shown in Figure 3A, primary cardiomyocytes cultured for 48 h exhibit normal morphology and appropriate cell confluence, and α-Actin staining was used to assess the purity of the primary cardiomyocytes. We further isolated exosomes secreted by primary cardiomyocytes and characterized their biological properties. TEM revealed that the extracted vesicles were uniform in morphology, elliptical in shape, with a lipid bilayer structure on the periphery and electron-low-density material inside, ranging from 30 to 200 nm in diameter (Figure 3B). NTA results showed that the vesicles exhibited Brownian motion in solution, as indicated by the dynamic light scattering signals (Figure 3C). Tracking and analysis of the Brownian motion revealed that the vesicle sizes were predominantly around 146.10 nm (Figure 3D). This confirms that the isolated vesicles in this study are cardiomyocyte-derived exosomes.

PKH67-labeled exosomes were injected into rats via the tail vein at a dose of 1.0 mg/kg. One hour later, the rats were euthanized, and their organs were collected. Fluorescence expression in the organs was assessed using the Bruker multimodal small animal imaging system FX Pro. After administration, the rats showed varying degrees of fluorescence expression in all organs, with the most pronounced fluorescence observed in the brain (Figure 3E).This suggests that cardiomyocyte-derived exosomes can circulate throughout the body and cross the blood-brain barrier to accumulate in the brain.

PKH26-exosomes at a concentration of 3.0 μg/mL were co-incubated with SH-SY5Y cells. As shown in Figure 3F, the PKH26-exosomes exhibit green fluorescence under fluorescence microscopy. After co-incubation for 12 h, numerous punctate green fluorescence signals are observed within the cytoplasm of the cells, while the PKH26 dye alone shows either no or minimal residual fluorescence. This indicates that cardiomyocyte-derived exosomes are internalized by SH-SY5Y cells. After the internalization, PCR was used to measure the expression of miR-181a-5p in SH-SY5Y cells (Figure 3G). MA, Con + Exo 1.5 μg/mL, Con + Exo 3.0 μg/mL, MA + Exo 1.5 μg/mL, and MA + Exo 3.0 μg/mL groups exhibited a significant increase in miR-181a-5p expression compared to the control group (all *P* < 0.01). Additionally, the MA + Exo 1.5 and 3.0 μg/mL groups exhibited a significant increase in miR-181a-5p expression compared to the MA group (all *P* < 0.01). This indicates after exosomes accumulate in the brain, they can be internalized by neurons and increase miR-181 expression in these cells.

### 3.3 Cardiomyocyte-derived exosomes can exacerbate MA-induced CPP effect

**FIGURE 4:**
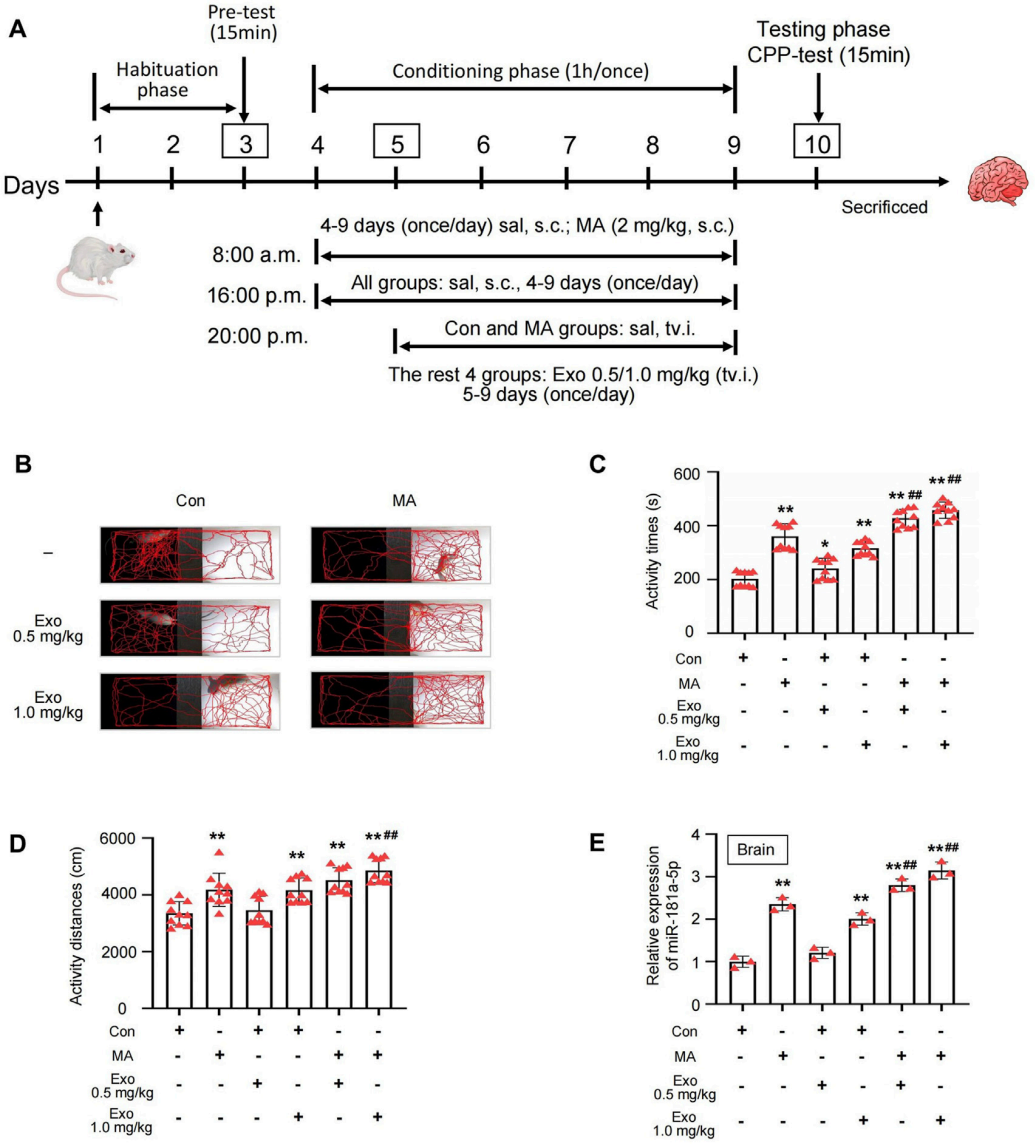
Exosomes can exacerbates MA-induced CPP effect. **(A)** Schematic protocol of the model of MA-dependent rats established by CPP. **(B)** Activity trajectories of rats in CPP box. **(C,D)** Activity time and distances of rats in white side of CPP box, n = 10 per group. **(E)** PCR detected the expression of miR-181a-5p in brain of rats which were induced with MA and received tail vein injections of exosomes, n = 3 per group. ^**^p < 0.01, ^*^p < 0.05 versus the control group; ^##^p < 0.01, ^#^p < 0.05 versus the MA model group. MA, methamphetamine; Con, Control; Exo, Exosome; sal, saline; s.c., subcutaneous injection; tv.i., tail vein injection.

We administered cardiomyocyte-derived exosomes via tail vein injection to investigate their effects on the CPP model of MA-dependent rats (Figure 4A). In the CPP experiment, the activity trajectory of six groups following CPP training is shown in Figure 4B, and MA, Con + Exo 1.0 mg/kg, MA + Exo 0.5 mg/kg, and MA + Exo 1.0 mg/kg groups demonstrated a marked increase in activity trajectories within the white box compared to the control group. Quantitative analysis revealed MA, Con + Exo 1.0 mg/kg, MA + Exo 0.5 mg/kg, and MA + Exo 1.0 mg/kg groups showed a significant increase in activity time and distance compared to the control group (all *P* < 0.01). MA + Exo 1.0 mg/kg group also showed a significant increase in activity time (*P* < 0.01) and distance (*P* < 0.01) compared to the MA group. And the MA + Exo 0.5 mg/kg group showed a significant increase in activity time (*P* < 0.01) compared to the MA group (Figures 4C,D).

After the CPP experiment, the rats were euthanized, and brain tissues were collected, and PCR was used to assess the expression levels of miR-181a-5p in the brains of each group (Figure 4E). MA, Con + Exo 1.0 mg/kg, MA + Exo 0.5 mg/kg, and MA + Exo 1.0 mg/kg groups exhibited a significant increase in miR-181a-5p expression compared to the control group (all *P* < 0.01). Additionally, the MA + Exo 0.5 and 1.0 mg/kg groups exhibited a significant increase in miR-181a-5p expression compared to the MA group (*P* < 0.01). These results suggest that the increasing dose of exosomes containing miR-181a-5p exacerbates MA CPP behavior of rats.

### 3.4 Network pharmacology analysis of miR-181a-5p

**FIGURE 5:**
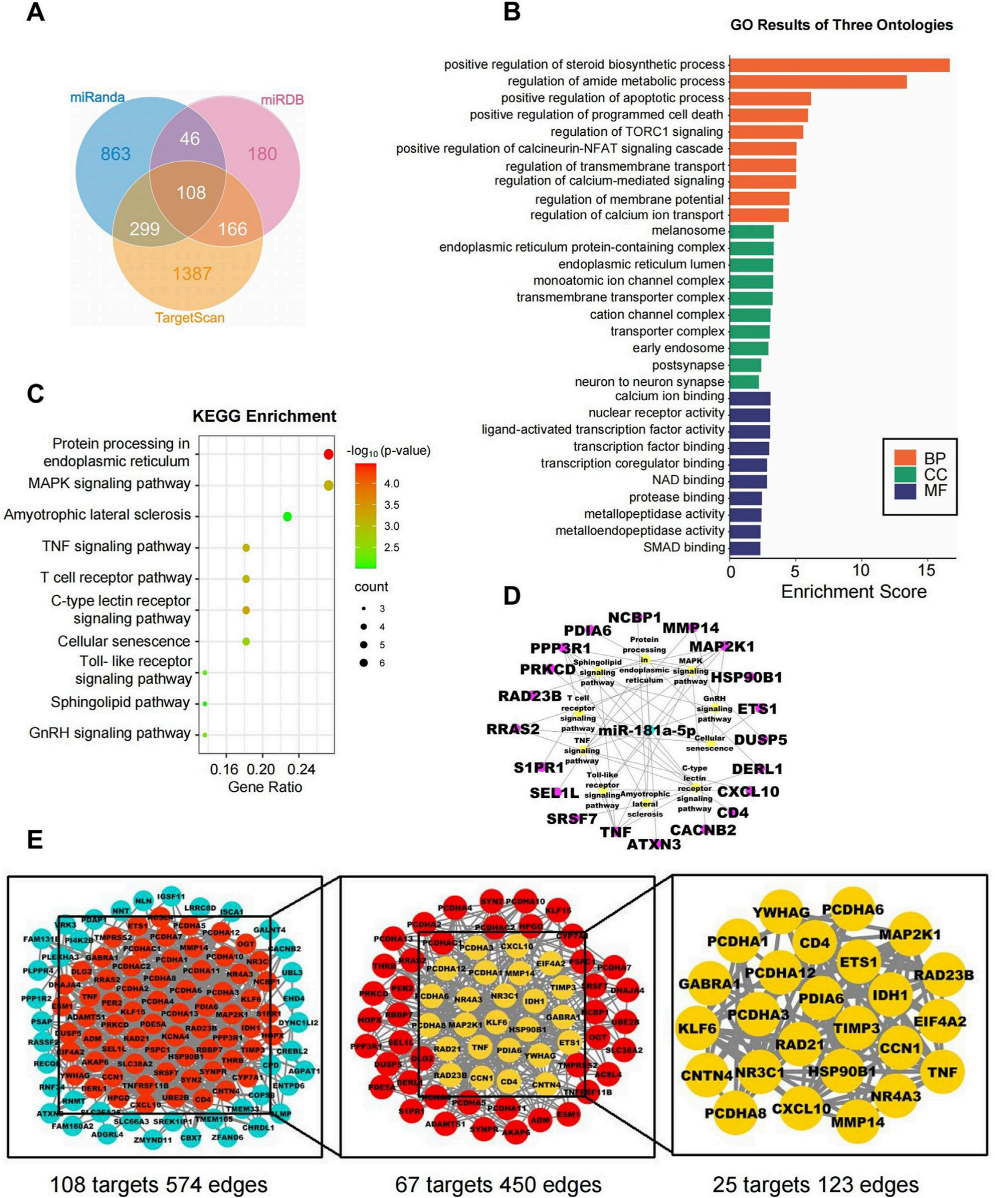
Network pharmacology analysis of miR-181a-5p. **(A)** The target genes of miR-181a-5p were predicted using three target gene prediction databases (Target Scan, miRDB and miRanda). **(B)** GO enrichment analysis. **(C)** KEGG enrichment analysis. **(D)** miR-181a-5p-pathway-target network. **(E)** miR-181a-5p key target PPI network.

Potential targets of miR-181a-5p were predicted by Target Scan, miRDB, and DIANA Tools, yielding 1960, 500, and 515 targets, respectively, with an overlap of 108 (Figure 5A; Table 3). The top ten GO terms are shown in Figure 5B. In the BP analysis, the target genes were largely enriched in positive regulation of steroid biosynthetic process, regulation of amide metabolic process, and positive regulation of apoptotic process; in the CC analysis, melanosome, endoplasmic reticulum protein-containing complex, and monoatomic ion channel complex were shown to be involved, while in the MF analysis, calcium ion binding, nuclear receptor activity, and ligand-activated transcription factor activity were discovered to be involved.

**TABLE 3:** The candidate target genes of miR-181a-5p.

Candidate target genes of miR-181a-5p								
Fam131b	Plekha3	Ppp3r1	Ubl3	Lrrc8d	Nr4a3	Ehd4	Cops8	Isca1
Slc25a25	Pcdhac1	Rassf2	Nnt	Akap6	Srsf7	Klf15	Eltd1	Clmp
Tmem165	Pcdha10	Pcdha2	Adm	Vrk3	S1pr1	Synpr	Psap	Hopx
Slc38a2	Entpd6	Map2k1	Ogt	Prkcd	Ube2b	Rnmt	Cbx7	Nr3c1
Pcdhac2	Srek1ip1	Pcdha7	Per2	Kcna4	Atxn3	Pde5a	Dnaja4	Ywhag
Dync1li2	Hsp90b1	Cyp7a1	Recql	Dusp5	Syn2	Sel1l	Ppp1r2	Cpd
Fam160a2	Pi4k2b	Pcdha3	Rras2	Galnt4	Ncbp1	Hpgd	Cntn4	Igsf11
Tmem168	Pcdha12	Pcdha4	Tnf	Pdia6	Pqlc3	Eif4a2	Derl1	Timp3
Pcdha11	Rad23b	Pcdha6	Cyr61	Rbbp7	Thrb	Rad21	Pspc1	Lppr4
Zmynd11	Pcdha13	Cacnb2	Acsl4	Pcdha8	Esm1	Crebl2	Rnf34	Cxcl10
Tnfrsf11b	Tmem33	Pcdha5	Chrdl1	Agpat1	Ets1	Nln	Dlg2	Cd4
Tmprss2	Adamts1	Mmp14	Idh1	Gabra1	Zfand6	Klf6	Pcdha1	Pdap1

KEGG pathway enrichment analysis was performed on the 108 intersecting targets and yielded a total of 22 related pathways, mainly including protein processing in the endoplasmic reticulum, the MAPK signaling pathway, and amyotrophic lateral sclerosis. The top ten KEGG pathways are shown in Figure 5C; additionally, a network was constructed between miR-181a-5p, the top ten signaling pathways, and their corresponding targets, as illustrated in Figure 5D.

The targets were screened with a medium confidence of 0.9, which resulted in 108 targets and 574 edges. The first screening with thresholds of Degree >10.00, Between >34.45, and Closeness >0.28 yielded 67 targets and 450 edges. With Degree >21.00, Between >111.62, and Closeness >0.38 as screening thresholds for the second screening, 25 targets and 123 edges were found for further analysis (Figure 5E). Among them, 12 high-degree targets (HSP90B1, RAD21, MMP14, TNF, TIMP3, PRKCD, PDIA6, YWHAG, RAD23B, MAP2K1, EIF4A2, CD4) are indicated to play a crucial role in the involvement of miR-181a-5p in drug addiction.

### 3.5 Molecular docking between 12 key targets of miR-181a-5p and MA

**FIGURE 6:**
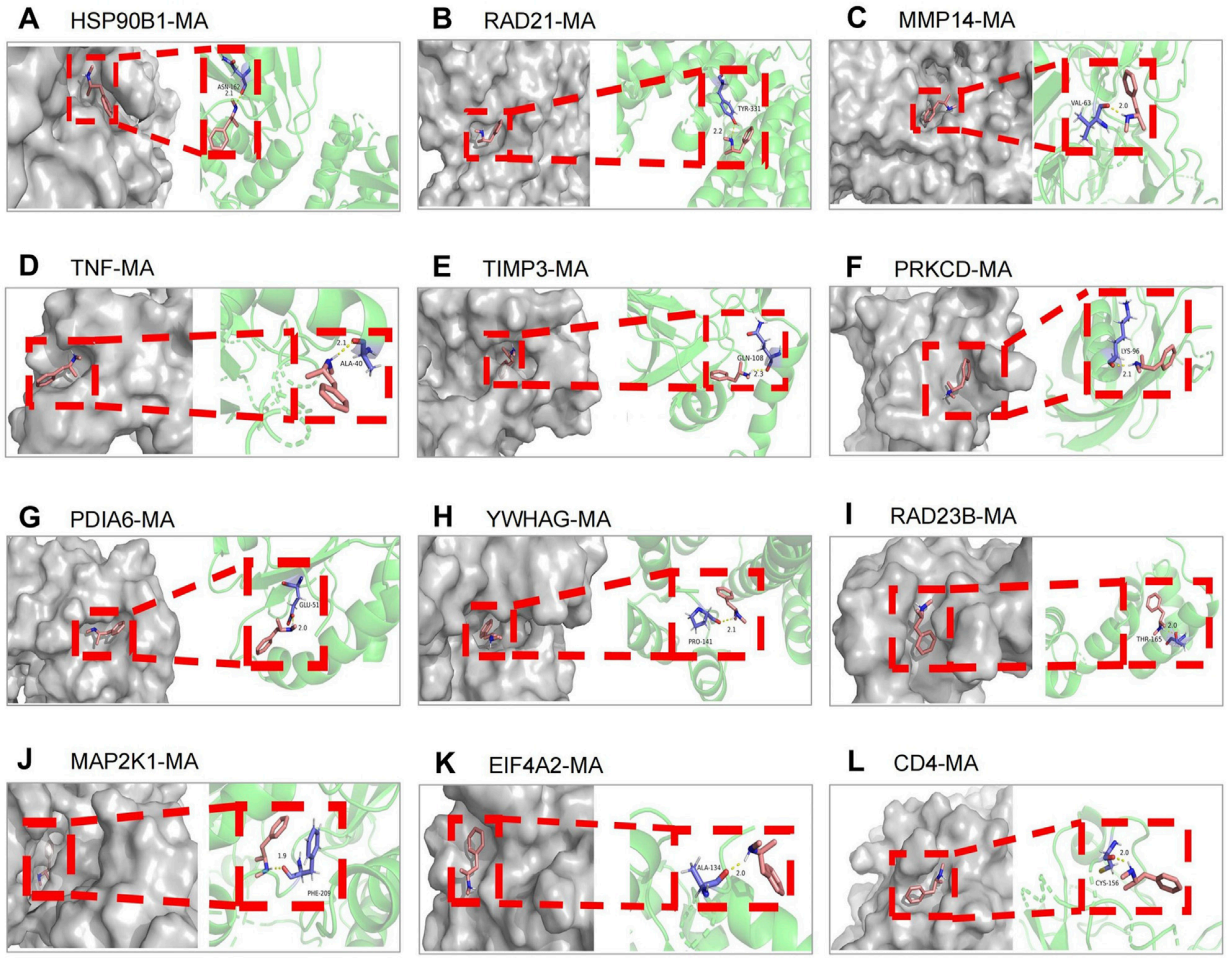
Molecular docking between 12 key targets of miR-181a-5p with MA. **(A–L)** Target genes in the following order: HSP90B1, RAD21, MMP14, TNF, TIMP3, PRKCD, PDIA6, YWHAG, RAD23B, MAP2K1, EIF4A2, CD4.

MA was selected as the ligand molecule, with the ligand’s mol2 file obtained from the PubChem database. The molecular rigid receptors for the 12 core targets (HSP90B1, RAD21, MMP14, TNF, TIMP3, PRKCD, PDIA6, YWHAG, RAD23B, MAP2K1, EIF4A2, and CD4) were retrieved from UniProt, and the corresponding pdbqt files were downloaded. These macromolecular receptor files were preprocessed in PyMOL for molecular docking, and the docking was performed using AutoDock 4.2.6. Docking images were generated with PyMOL (Figures 6A–L), showing stable interactions between MA and the 12 core targets of miR-181a-5p. The combined docking energies for MA and the 12 core targets are summarized in Table 4.

**TABLE 4:** Basic information of molecular docking of MA and key targets.

Targets	Compound	PDB number	Combined energy (kcal· mol^-1^)
HSP90B1	MA	7ull	−3.86
RAD21	MA	6rrc	−3.92
MMP14	MA	4p3d	−4.64
TNF	MA	6q00	−5.22
TIMP3	MA	3cki	−4.13
PRKCD	MA	1yrk	−4.54
PDIA6	MA	3vww	−4.96
YWHAG	MA	6s9k	−4.54
RAD23B	MA	6w2g	−4.59
MAP2K1	MA	7b7r	−4.18
EIF4A2	MA	3bor	−3.73
CD4	MA	2xu3	−5.57

### 3.6 Dual-luciferase validate the binding sites between miR-181a-5p and 6 key targets

**FIGURE 7:**
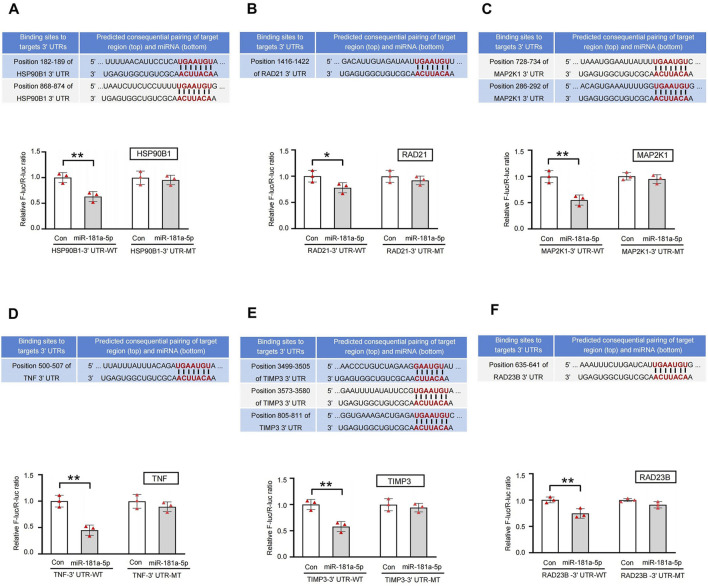
Sequence alignment of miR-181a-5p with the 3′-UTR of 6 key targets gene and validation of dual-luciferase reporter assay. **(A–F)** Target genes in the following order: HSP90B1, RAD21, MAP2K1, TNF, TIMP3, RAD23B. miR-181a-5p mimic reduced the activity of luciferase linked to the WT 3′-UTR of targets gene, and the MT 3′-UTR of targets gene eliminated the mimic’s effect on the activity of luciferase in HEK 293T cells, n = 3 per group. ^**^p < 0.01, ^*^p < 0.05 versus the control group. Con, control group.

Analysis by Gene Cards revealed that HSP90B1, RAD21, MAP2K1, TNF, TIMP3, and RAD23B contain highly conserved binding sites for miR-181a-5p. Dual-luciferase reporter assays demonstrated that the miR-181a-5p mimic significantly reduced luciferase activity of HSP90B1, RAD21, MAP2K1, TNF, TIMP3, and RAD23B linked to the WT 3′-UTR, and MT 3′-UTR completely abolished the mimic’s effect (*P* < 0.05 or *P* < 0.01), indicating that miR-181a-5p negatively regulates these six targets by specifically binding to their 3′-UTR sequences (Figures 7A–F).

## 4 Discussion

**FIGURE 8:**
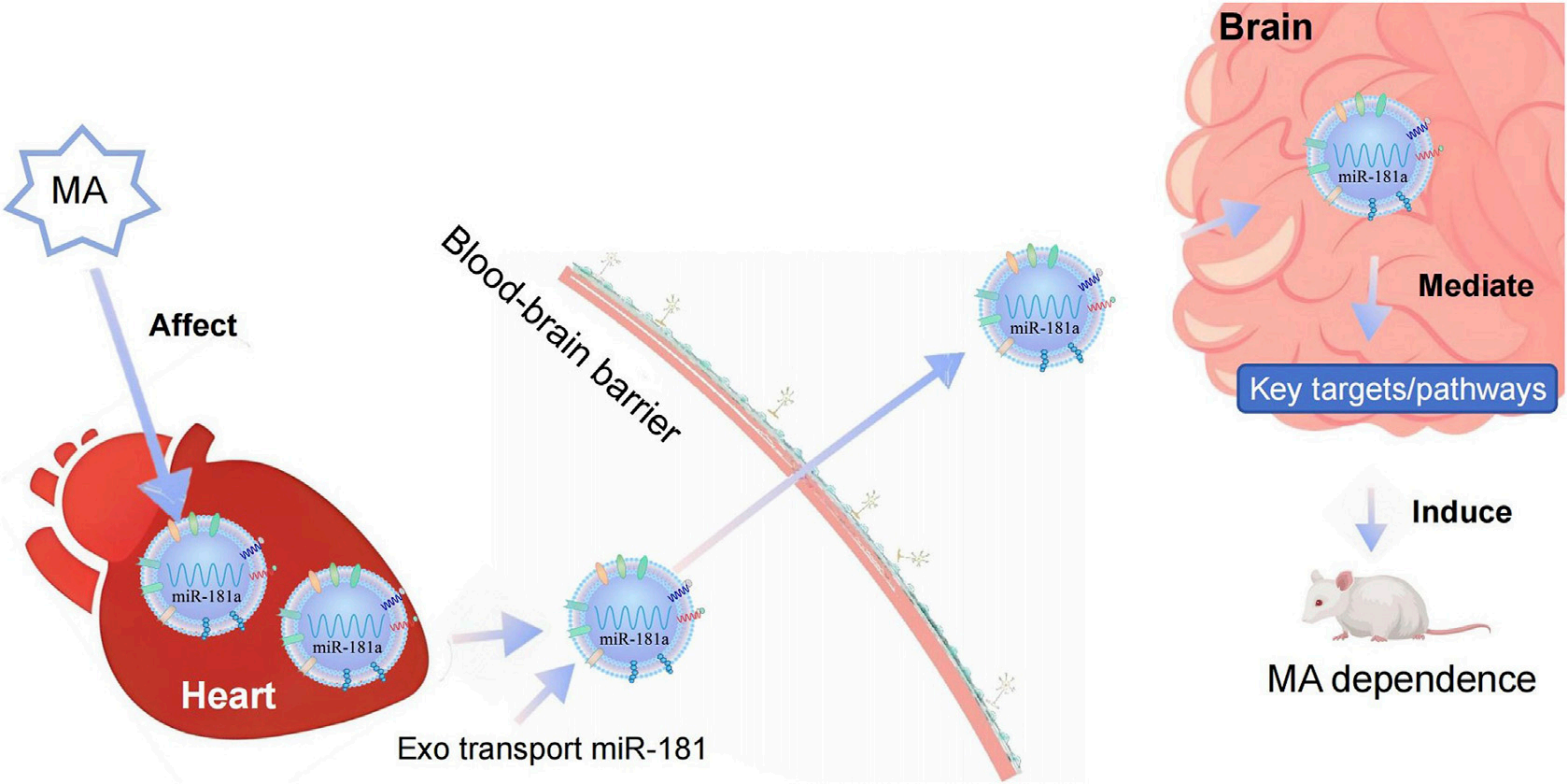
Diagram illustrating the biological mechanisms of cardiac exosome-mediated heart-brain crosstalk in MA dependence. MA, methamphetamine; Exo, Exosome.

The present study demonstrates that MA induces CPP in rats and triggers an upregulation of miR-181a-5p in both cardiomyocyte-derived exosomes and the brain. These exosomes, carrying miR-181a-5p, play a pivotal role in mediating heart-brain crosstalk. They circulate throughout the body, cross the BBB, and are internalized by SH-SY5Y cells, where they increase miR-181a-5p expression. Importantly, cardiomyocyte-derived exosomes containing miR-181a-5p exacerbate MA-induced CPP behaviors, suggesting a novel mechanism through which the heart contributes to the central effects of MA via exosome-mediated miRNA transfer. Network pharmacology analysis identified 108 potential targets of miR-181a-5p, and molecular docking revealed stable interactions between MA and 12 of these targets. Dual-luciferase reporter assays confirmed that miR-181a-5p specifically binds to the 3′-UTR sequences of six of these targets, downregulating their expression. Based on the *in vivo*, *in vitro*, and network pharmacology experiments described above, we propose that during MA dependence, cardiomyocyte-derived exosomes can carry miR-181a-5p, then circulate through the bloodstream and accumulate in the brain. Once in the brain, the exosomal miR-181a-5p can further regulate its target genes and downstream signaling pathways, thereby influencing MA-dependent behaviors. Exosomes as mediators of peripheral-central communication and exacerbate MA dependence in rats (Figure 8).

Studies have shown that overexpression of miR-1 in the hearts of mice leads to the transfer of miR-1 via exosomes to the central hippocampus. This transfer further regulates the transcription of synaptosome-associated protein 25, thereby reducing synaptic vesicle exocytosis in the hippocampus. This research reveals that the heart can communicate with the central nervous system through exosomes, serving as a unique carrier for heart-brain crosstalk (Duan et al., 2018). In PD research, exosomes derived from blood have demonstrated inherent brain-targeting capabilities. These exosomes successfully deliver dopamine to the brain, including the striatum and substantia nigra. When blood-derived exosomes were used as a delivery system, dopamine distribution in the brain increased more than 15-fold. Exosomes loaded with dopamine showed better therapeutic efficacy and lower systemic toxicity compared to intravenous administration of free dopamine in PD mouse models, suggesting that blood-derived exosomes can be used to deliver targeted dopamine therapy for PD and other central nervous system diseases (Mengke et al., 2016). Exosomes can transfer miRNAs, thus playing a role in disease progression. The functions of exosomes secreted by different cell types vary, and the same cell may secrete exosomes with distinct roles under different pathological conditions. The key factor lies in the biological functions and properties of the miRNA, mRNA, and specific proteins encapsulated within the exosomes.

MiRNAs are known to regulate various aspects of brain development, neuronal differentiation, and gene expression in neurons (Christensen and Schratt, 2009). Elevations in miR-181a levels are associated with synaptic dysregulation and metabolic damage. Conversely, downregulation of miR-181 has been shown to reduce oxidative stress, neuronal death, and neuroinflammation (Kong et al., 2020). Recent studies suggest that miR-181a-5p is implicated in 126 gene targets related to neurological and psychological disorders. In this study, miR-181a-5p was significantly overexpressed in the nucleus accumbens of rats exhibiting chronic MA-induced CPP, which correlates with MA addiction phenotypes (Sim et al., 2017). Furthermore, a recent report demonstrated that miR-181a modulates MA-induced attention deficit disorder through the miR-181/SIRT1 axis (Rashidi et al., 2024). Another study identified miR-181a′s involvement in cocaine-responsive plasticity genes within the mesolimbic dopaminergic system under chronic cocaine exposure (Chandrasekar and Dreyer, 2009). Our findings, combined with previous work, suggest that miR-181a-5p may be essential in modifying synaptic functions in the hippocampus or nucleus accumbens following MA exposure, ultimately contributing to the formation of MA dependence (Jiang et al., 2023). These observations emphasize miR-181a-5p′s potential role in drug-induced neuroplasticity, particularly in the nucleus accumbens, a critical brain region for addiction (Sim et al., 2017). A deeper understanding of its role could pave the way for novel therapeutic strategies for managing substance use disorders.

Our study identified 6 key targets of miR-181a-5p, including HSP90B1, RAD21, MAP2K1, TNF, TIMP3, and RAD23B. These targets are involved in crucial biological processes relevant to addiction and neurobiology. For example, in HD, HSP90B1 is linked to the pathogenic gene, influencing protein aggregation and toxicity (Kalathur et al., 2015). RAD21 is involved in DNA repair and gene transcription regulation (Bianco et al., 2018). Studies have shown that RAD21 interacts with opioid-binding cell adhesion molecules to mediate neuroprotection in PD models (Zhang et al., 2022). Furthermore, RAD21 plays a role in promoting vascular endothelial growth factor A and protecting against neuronal apoptosis in models of acute spinal cord injury (Li et al., 2021; Wang et al., 2023). TNF-α levels have been shown to be significantly elevated in drug-abusing individuals (Luo et al., 2022), and its presence in astrocytes in the hippocampus of MA-exposed mice suggests a neuroinflammatory component to MA addiction (Canedo et al., 2021). TIMP3, a neuroprotective protein, enhances neuronal survival and outgrowth, with potential therapeutic effects in traumatic brain injury (Gibb et al., 2015). RAD23B is involved in DNA repair and neurodegeneration in neurodegenerative diseases such as amyotrophic lateral sclerosis (Wang et al., 2019; Schludi et al., 2017), while MAP2K1 plays a critical role in synaptic plasticity and neurotransmission in addiction and psychiatric disorders (Alasmari et al., 2020; O'Connell et al., 2023).

These 6 key targets of miR-181a-5p are involved in multiple biological processes relevant to addiction and neurobiology, underscoring their potential as therapeutic targets and their implication in a variety of neurological conditions, including addiction, neurodegeneration, and psychiatric disorders. Their complex interactions provide valuable insights into the molecular mechanisms of drug addiction, offering a foundation for further investigation into their role in addiction and as therapeutic candidates. The present study has provided new insights into the role of cardiomyocyte-derived exosomes carrying miR-181a-5p in MA dependence. Our results suggest that the heart and brain communicate through exosome-mediated transfer of miRNAs, exacerbating MA-induced CPP behaviors in rats and offering a novel perspective on the peripheral-central interactions in MA addiction. By employing a combination of behavioral assessments, molecular biology techniques, nanotechnology, network pharmacology, and bioinformatics, we have explored the mechanism of MA dependence at multiple levels. Network pharmacology analysis and molecular docking experiments identified 108 potential targets of miR-181a-5p, with six confirmed targets interacting directly with the miRNA. These findings highlight the importance of these targets in addiction-related biological processes, providing a comprehensive understanding of the molecular mechanisms of MA addiction.

This study provides valuable insights into the molecular mechanisms underlying MA dependence, particularly highlighting the role of cardiomyocyte-derived exosomes carrying miR-181a-5p in facilitating heart-brain communication. Previous studies have demonstrated that the concentration of miRNAs in exosomes is significantly higher than that within the brain tissue itself (Fang et al., 2022). In the present study, miR-181a-5p was detected in both the brain and cardiomyocyte-derived exosomes, with markedly higher levels observed in the exosomes. Following tail vein injection, exosomes were found to accumulate in the brain, accompanied by a significant upregulation of miR-181a-5p expression compared to baseline levels. These findings suggest that during MA exposure, cardiac exosomes may serve as carriers that deliver miR-181a-5p to the brain, thereby playing a pivotal role in mediating the observed effects.

While the CPP model effectively assesses drug reward and associative learning, it does not evaluate relapse or drug-seeking behaviors, which are critical aspects of addiction pathology. Moreover, this study did not investigate region-specific accumulation of exosomes within the brain. Additionally, exosome-specific protein markers were not confirmed via western blotting, and the cardiac origin of the exosomes was not experimentally verified. In the extracellular uptake assays, the cultured cells were not treated with MA, which may limit the translational relevance of the findings. Finally, this study did not employ miR-181a-5p inhibitors to determine whether blocking its function could attenuate CPP. Addressing these limitations is a priority for future studies investigating the molecular mechanisms of MA dependence.

## 5 Conclusion

This study reveals that cardiomyocyte-derived exosomes enriched with miR-181a-5p facilitate heart-brain crosstalk and exacerbate MA dependence in rats. miR-181a-5p modulates key addiction-related signaling pathways, presenting a promising therapeutic target for mitigating MA addiction through peripheral-central nervous system interactions. Future studies should further investigate the translational potential of exosome-based therapies and integrative models in the treatment of substance dependence.

## Data Availability

The original contributions presented in the study are included in the article/supplementary material, further inquiries can be directed to the corresponding authors.

## Abbreviations

MA, Methamphetamine; CNS, central nervous system; BBB, blood-brain barrier; PD, Parkinson disease; AD, Alzheimer disease; SD, Sprague-Dawley; CPP, conditioned positional preference; TEM, Transmission electron microscopy; NTA, Nanoparticle tracking analysis; s.c., subcutaneous; tv.i., tail vein injection; BP, biological process; CC, cellular component; MF, molecular function; PPI, protein-protein interaction; DAVID, database for annotation, visualization and integrated discovery; GO, Gene Ontology; KEGG, Kyoto Encyclopedia of Genes and Genomes; PDB, protein data bank; WT, wild-type; MT, mutant-type; 3′-UTRs, 3′-untranslated regions.
